# Light Harvesting Proteins for Solar Fuel Generation in Bioengineered Photoelectrochemical Cells

**DOI:** 10.2174/1389203715666140327105530

**Published:** 2014-06

**Authors:** Julian Ihssen, Artur Braun, Greta Faccio, Krisztina Gajda-Schrantz, Linda Thöny-Meyer

**Affiliations:** 1 Laboratory for Biomaterials, Empa, Swiss Federal Laboratories for Materials Science and Technology, CH – 9014 St. Gallen, Switzerland;; 2 Laboratory for High Performance Ceramics, Empa, Swiss Federal Laboratories for Materials Science and Technology, CH – 8600 Dübendorf, Switzerland;; 3 University of Szeged, Department of Inorganic and Analytical Chemistry, H-6701 Szeged, Hungary

**Keywords:** Artificial photosynthesis, hematite, light harvesting, photoelectrochemical cell, photosensitizer, phycocyanin, protein immobilization, solar hydrogen.

## Abstract

The sun is the primary energy source of our planet and potentially can supply all societies with more than just their basic energy needs. Demand of electric energy can be satisfied with photovoltaics, however the global demand for fuels is even higher. The direct way to produce the solar fuel hydrogen is by water splitting in photoelectrochemical (PEC) cells, an artificial mimic of photosynthesis. There is currently strong resurging interest for solar fuels produced by PEC cells, but some fundamental technological problems need to be solved to make PEC water splitting an economic, competitive alternative. One of the problems is to provide a low cost, high performing water oxidizing and oxygen evolving photoanode in an environmentally benign setting. Hematite, α-Fe_2_O_3_, satisfies many requirements for a good PEC photoanode, but its efficiency is insufficient in its pristine form. A promising strategy for enhancing photocurrent density takes advantage of photosynthetic proteins. In this paper we give an overview of how electrode surfaces in general and hematite photoanodes in particular can be functionalized with light harvesting proteins. Specifically, we demonstrate how low-cost biomaterials such as cyanobacterial phycocyanin and enzymatically produced melanin increase the overall performance of virtually no-cost metal oxide photoanodes in a PEC system. The implementation of biomaterials changes the overall nature of the photoanode assembly in a way that aggressive alkaline electrolytes such as concentrated KOH are not required anymore. Rather, a more environmentally benign and pH neutral electrolyte can be used.

## INTRODUCTION

Mankind is in urgent need of replacing fossil energy sources by renewable forms of energy. Theoretically, the sunlight which reaches the earth’s surface easily satisfies current and future human consumption of energy. The power of 1 h of sunlight falling on our planet is equivalent to the current annual global consumption of approximately 16.3 TW [1]. Therefore, the direct conversion of solar radiation into technically useful forms of energy such as fuels and electricity is the most elegant, but also the most challenging route towards renewables. The generation of electricity by photovoltaic (PV) devices has emerged as a readily available, sustainable technology with a steadily increasing market share. It has been estimated that an area of 100 x 100 km covered with PV modules with a power conversion efficiency of 15% could satisfy the electricity demand of the United States of America [2]. However, the global consumption of energy stored in the form of chemical bonds (fuels) exceeds that of electricity, and for the conversion of sunlight into fuels, a viable technology is still missing, in spite of the fact that this topic has increasingly attracted scientific attention.

Natural photosynthesis has for a long time been an inspiration for solving mankind’s energy supply problems. Photosynthetic organisms convert solar photons into usable chemical energy by performing a water splitting reaction coupled to the synthesis of carbohydrates from atmospheric CO_2_. The photosynthetic apparatus of plants, algae and photosynthetic bacteria has been investigated in great detail. It is composed of a complex membrane-associated arrangement of light harvesting proteins, photosynthetic reaction centers, electron transport proteins and ATP synthase [3-5]. The observed natural structures of thylakoid membranes and chloroplasts suggest that rather than single molecules - irrespective of their molecular complexity - hierarchical macromolecular architectures are necessary to perform photosynthetic energy conversion. This is reflected in a statement of one of the most influential scientists in photosynthesis research, Melvin Calvin: “…*The primary quantum conversion act… cannot occur in isolated charge transfer molecules in solution because the products cannot escape from each other. … It can only take place in a laminated structure where the electrons and holes can escape from each other by electron migration and not by atomic migrations*” [6]. Artificial photosynthetic devices attempt to mimic biological systems. However, natural photosynthesis, when considering the overall growth process of photosynthetic organisms, is not very efficient. Only 3–4% of the total sunlight energy falling onto a leaf is converted into chemical energy, while current silicone and gallium-arsenide PV cells reach efficiencies well above 20% (conversion efficiency of solar to electrical energy, η), corresponding to photocurrent densities of 20-40 mA cm^-2^ [7, 8]. Therefore, the ultimate goal is to develop artificial photosynthetic devices which surpass the efficiency of natural systems.

**Fig. (1):**
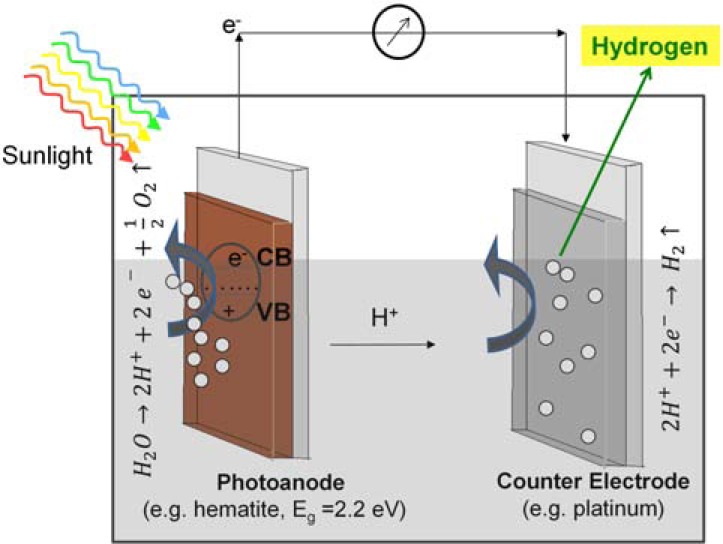
Principle of a PEC cell. Light induces a charge separation within the photoanode semiconductor (CB: conduction band, VB: valence band), which in turn leads to photocatalytic water splitting into H^+^ ions and molecular oxygen and the generation of electrons. The H^+^ ions migrate to the counter electrode (cathode) where they are reduced to H_2_ with the generated electrons. In most PEC cells, a certain external overpotential (bias) of 0.5-1 mV needs to be applied in order reach significant hydrogen production rates.

Artificial photosynthesis is currently taking center stage in the scientific world, also in Europe [9]. Photoelectrochemical (PEC) cells are artificial photosynthetic devices which increasingly receive attention since the turn of the millennium [10]. They produce hydrogen directly from sunlight by performing a water splitting reaction with semiconducting metal oxide electrodes. Similarly to photosynthetic reaction centers, a light driven charge separation occurs within the semiconductor photoelectrode, creating electron/electron-hole pairs. At the photoanode, the electron-holes reach the semiconductor surface and oxidize water, which leads to the formation of O_2_ and H^+^ at the semiconductor/liquid junction (Fig. **1**). The cathode is placed in the same electrolyte, thus allowing migration of H^+^ ions to the counter electrode and subsequent reduction to H_2_ (Fig. **1**). The cathode should be composed of a catalyst facilitating H_2_ formation, e.g. platinum [11]. It is also possible to use a photocathode, where photoelectrons diffuse to the electrolyte and reduce H^+^ to hydrogen. For PEC water splitting on iron oxide as shown in this paper, an external bias is needed in order to raise the conduction band to the water oxidation potential [12]. This overpotential can be provided by a coupled photovoltaic cell [12]. The required overpotential can be decreased by the Nernst potential of a strongly alkaline electrolyte such as KOH [12].

The suitability of solar cells (and PEC cells) for energy conversion depends on four essential criteria: (i) efficiency, (ii) stability, (iii) cost effectiveness and (iv) material availability [3]. For fabrication of PEC photoanodes, primarily metal oxide materials such as TiO_2_, WO_3_, and Fe_2_O_3_ have been investigated [12, 13]. PEC water splitting on TiO_2_ photoanodes is known since the 1970s [11]. However, only the UV part of the sunlight can be utilized in such PEC cells due to the large band gap of this semiconductor (*E_g_* =3.2 eV). In contrast, hematite (α-Fe_2_O_3_) has a smaller band gap of 1.9 to 2.2 eV and strongly absorbs yellow to ultraviolet photons also in the visible region of 300-600 nm [12, 14]. Theoretically, 16.8% of the sun’s energy can be converted to H_2_ with this material [12]. Hematite is a very promising PEC material also with respect to criteria (ii) to (iv): iron oxides belong to the most abundant materials on the earth’s surface, are non-toxic and stable in the present-day oxic atmosphere [12, 13]. Hematite is also stable in strongly alkaline electrolytes which are typically applied in electrolyzer cells. An ongoing challenge is to push the efficiency of hematite-based PEC cells towards the theoretical limit. The poor electronic structure of hematite is major challenge, in particular the low efficiency of charge carrier transport (conductivity). By nanostructuring and silicon doping of hematite photoanodes it was possible to increase photocurrent density from below 0.2 to 3.3 mA cm^-2^ [12].

An efficient, low-cost material for the hydrogen-generating photocathode is currently lacking. Efforts have recently been made to replace the conventional platinum electrodes by metal hydride or copper oxide composite cathodes [15, 16]. Another option would be to use very thin platinum layers on a cheaper conducting material.

A new approach for boosting the H_2_ yield of hematite-based PEC cells, which again is inspired by natural photosynthesis, is the incorporation of light harvesting proteins/pigments with additional photon absorption capabilities. Here, hybrid bio-inorganic PV and PEC cells are reviewed, followed by a discussion of a particularly interesting class of photosynthetic proteins, the phycobiliproteins. Finally, we will delineate our efforts to generate efficient PEC cells by immobilizing the light harvesting protein c-phycocyanin on hematite thin films.

## PHOTOSYNTHETIC PROTEINS IN PHOTOVOLTAIC AND PHOTOELECTROCHEMICAL DEVICES

The combination of inorganic and organic materials for solar energy harvesting is the key feature of dye sensitized solar cells (DSSC) [17], a promising low-cost alternative to classical silicone PV cells [3, 8, 18]. In biomimetic or hybrid bio-inorganic PV and PEC devices, either light-harvesting proteins alone or whole photosynthetic reaction centers are used to “sensitize” metal and semiconductor surfaces [3, 19]. Table **1** gives an overview of different approaches for the fabrication of bio-functionalized photoanodes. The natural pigment phycocyanin (for details on this light harvesting protein see next section) was used for photosensitization of colloidal TiO_2_ nanoparticles [20]. Fluorescence lifetime measurements indicated that electrons from adsorbed excited state phycocyanin were directly injected into the conduction band of TiO_2_. In a related experiment, PEC photoanodes composed of TiO_2_ nanotubes were functionalized with the light harvesting protein bacteriorhodopsin from *Halobacterium salinarum *(Table **1**), which resulted in a 50% increase in efficiency under simulated solar illumination [21]. When the redox mediator pair iodide/tri-iodide was added to the electrolyte, photocurrent densities of up to 0.87 mA cm^-2^ could be reached.

Lu *et al.* adsorbed photosynthetic reaction centers from the photosynthetic bacterium *Rhodobacter sphaeroides* on a mesoporous WO_3_-TiO_2_ film [23]. In a PEC cell containing Tris∙HCl buffer with sodium dithionite as redox mediator, photocurrent density at a light intensity of 5 mW cm^-2 ^was increased from 6 to 30 µA cm^-2^ by this type of bio-functionalization (Table **1**). A biophotovoltaic device designed by Mershin *et al.* utilized self-assembling, genetically engineered PSI systems which were immobilized on TiO_2_-ZnO photoanodes [24]. Photocurrent density at simulated solar light illumination reached up to 362 µA cm^-2^ (Table **1**). In a highly successful biofunctionalization study, photosystem I (PSI) complexes were extracted from commercially available spinach leaves and coated onto p-doped silicon electrodes [25]. A four-fold increased photocurrent density of 875 μA cm^2^ was achieved compared to bare silicon in the presence of 0.2 M of the redox mediator methyl viologen (Table **1**). Photosynthetic proteins can also be used for enhancing the performance of hematite photoanodes. In experiments performed in our own laboratory [14], photocurrents of up to 490 µA cm^2^ were reached with such a system without adding any redox mediator (Table **1**, described in detail in the following chapters).

Indium tin oxide (ITO) semiconducting electrodes were also functionalized with photosynthetic proteins, but photocurrent densities were lower than for TiO_2_ and hematite based systems. The best-performing bio-hybrid ITO electrode was developed by Kato *et al.* who integrated cyanobacterial photosystem II (PSII) from *Thermosynechococcus elongatus* in a mesoporous film of this material [26]. The three-dimensional metal oxide environment allowed for high protein coverage and the maximal photocurrent densities achieved with this PEC system were 1.6 μA cm^-2^ without redox mediator and 22 μA cm^-2^ with the mediator 2,6-dichloro-1,4-benzoquinone added to the electrolyte (Table **1**). When a monolayer of light-harvesting protein and reaction center structures of *Rhodospirillum rubrum* was formed on aminopropylsilane-activated ITO electrodes by self-assembly, a maximal photocurrent density of 0.9 nA cm^-2^ was obtained [28]. Tan *et al.* combined fluorine-doped tin oxide (FTO) glass electrodes with reaction center/antenna proteins from *Rhodobacter sphaeroides* [29]. The redox mediator N,N,N,N-tetramethyl-*p*-phenylene-diamine was used to shuttle electrons to a platinum-coated counter electrode. In this case, a photocurrent density of 0.9 µA cm^-2^ was achieved.

Numerous studies describe the functionalization of (noble) metal electrodes with photosynthetic proteins, but the obtained photocurrent densities were one to two orders of magnitude lower than for semiconductor electrodes and mostly ranged from 0.1 to 1.5 µA cm^-2^ mW^-1^ [19]. One study directly compared semiconductor and metal electrodes. Spinach PSI yielded a more than 2000-fold lower current density of only 0.35 μA cm^-2^ when coated onto gold electrodes instead of p-doped silicon [25]. In another type of bio-hybrid photoelectrochemical cell, multiple layers of spinach PSI were applied on a gold cathode, while the anode consisted of ITO coated plastic. The NaCl electrolyte had a neutral pH and contained the electron mediator dichloroindophenol. When this PEC cell was illuminated with 8 mW/cm^2^, the maximal photocurrent density was 8 μA cm^-2^ [30]. Similarly to the approach of Tan *et al.* for the sensitization of FTO electrodes [29], den Hollander *et al.* [31] used complexes of reaction centers and light harvesting proteins of *Rhodobacter sphaeroides *for the fabrication of a bio-hybrid device using bare gold electrodes. The maximal photocurrent density of this system was 25 μA cm^-2^. Photocurrent densities in a similar range were reached by Badura *et al.* who immobilized PSI extracted from thylakoid membranes of the cyanobacterium *T. elongatus* on gold electrodes by entrapment in crosslinked redox hydrogels (Table **1**) [27]. For the best-performing hydrogel containing an osmium complex the authors report a photocurrent density of 33 μA cm^-2^ at light saturation (Table **1**). Recently, also graphene electrodes were functionalized with spinach PSI, resulting in a 7-fold increased photocurrent of 0.55 µA cm^-2^ when illuminated with white light of an intensity of 82 mW cm^-2^ [32].

An interesting bio-hybrid system was described by Reisner *et al.* which extends the DSSC concept to the bio-functionalization of PEC cathodes [33]. TiO_2 _nanoparticles were first coated with a highly efficient ruthenium-containing sensitizer molecule (RuP) and then with a robust, oxygen tolerant bacterial [NiFeSe] hydrogenase. Upon excitation by visible light and in the presence of a sacrificial electron donor, H_2_ could be produced at a turnover rate of 50 (mol H_2_) s^-1^ (mol total hydrogenase)^-1^ at pH 7 and 25°C. Apparently, electrons generated by TiO_2_-catalysedwater oxidation were transferred directly to the adsorbed hydrogenase, which in turn reduced H^+^ from the buffered aqueous solution to H_2_. Functionalization of TiO_2_ with hydrogenase allowed the authors to bypass the use of expensive noble metals such as platinum for H_2_ generation.

## PHYCOBILIPROTEINS - ABUNDANT NATURAL PHOTOSENSITIZERS

**Fig. (2):**
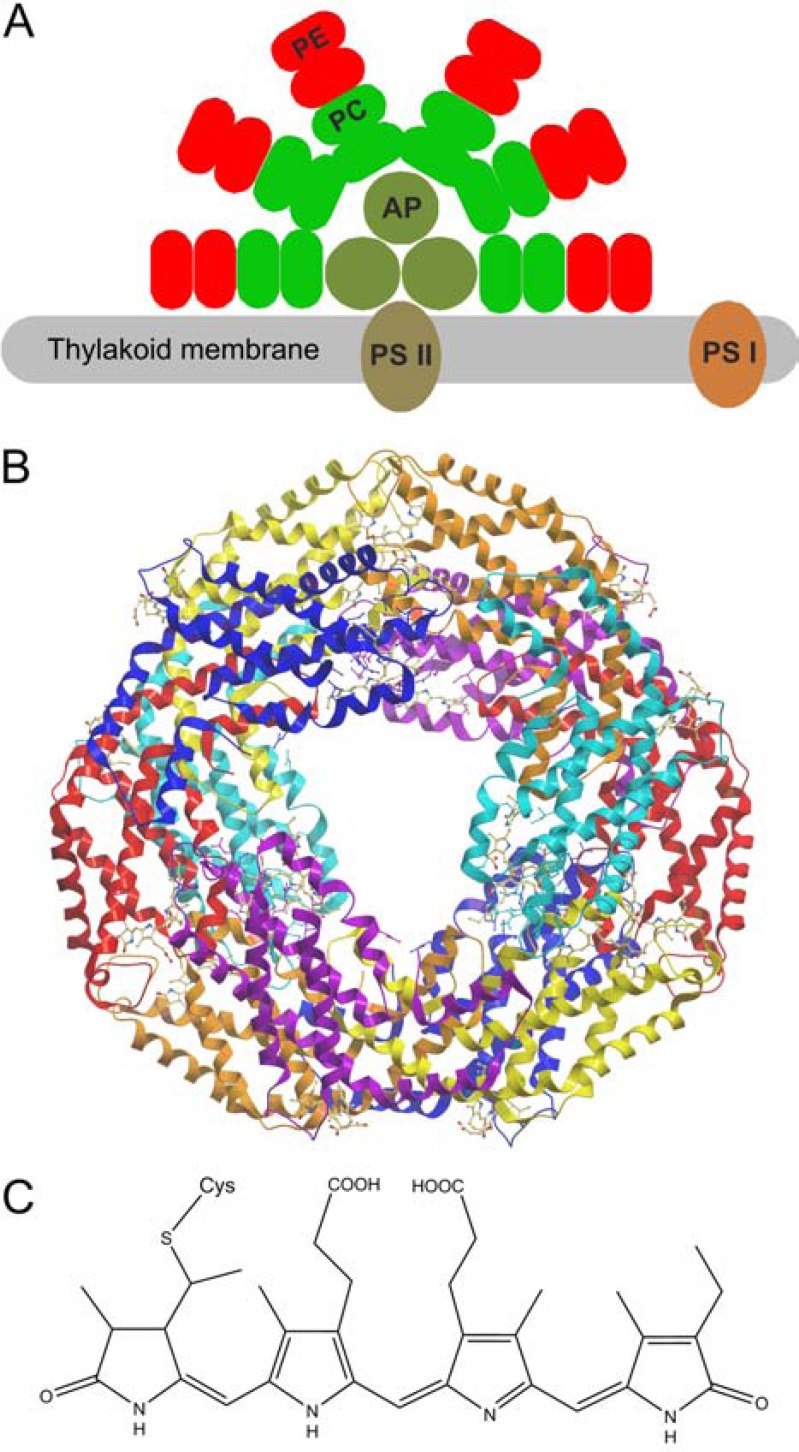
Structure of phycobiliproteins. (**A**) Simplified scheme of a cyanobacterial light harvesting complex (phycobilisome), PE: phycoerythrin, PC: phycocyanin, AP: allophycocyanin. (**B**) Crystal structure PDB No. 1GH0 of a c-phycocyanin hexamer of *Arthrospira platensis* [40]; α-subunits are colored red, yellow and orange, β-subunits are colored blue, cyan and violet, phycocyanobilin co-factors are depicted as atom-colored ball-stick representations. (**C**) Chemical structure of the phycocyanobilin chromophore in the open form, attached to the cysteine of the carrier protein.

Phycobilisomes are crucial components of the photosynthesis apparatus of prokaryotic cyanobacteria, formerly called “blue-green algae”, and of two eukaryotic algal genera, Rhodophyta (“red algae”) and Cryptophyta. These multi-protein-pigment complexes are composed of several types of light harvesting proteins, the phycobiliproteins and photosystem II (PSII), and reside in the thylakoid membrane (Fig. **2A**). The light harvesting “antenna” consists of multiple layers of allophycocyanin (APC, A_max_ ≈ 650 nm) at the core, phycocyanin (PC,*A*_max_ ≈ 620 nm) within the rods, and in some species also phycoerythrin at the tip of the rods (PE, *A*_max_ ≈ 560 nm) (Fig. **2A**) [34-36]. All three spectroscopically distinct phycobiliproteins have a common subunit organization [37]. A heterodimer composed of an α- and β-polypeptide chain is the basic unit. Each of the monomeric subunits consists of nine a-helical domains separated by flexible loops. Three dimers form a disc-shaped (αβ)_3_ trimer. Finally a (αβ)_6_ hexamer results from the tight association of two trimers. Multiple stacks of hexamer discs make up the phycobilisome rods which are connected to PSII by linker proteins. The crystal structure of phycobiliproteins is known since 1985 when the first structure of a cyanobacterial PC was published [38]. The structure of PE was first resolved by Ficner *et al.* in 1992 [39]. Since then, the crystal structures of phycobiliproteins from diverse species have been determined. As an example the structure of c-phycocyanin from the cyanobacterium *Athrospira platensis* (syn.* Spirulina platensis*) is shown in (Fig. **2B**) [40]. Both the α- and the β-subunit contain tetrapyrrole chromophores (bilins) that are covalently linked to the polypeptide chain via thioether bonds at conserved cysteine residues by specific phycobilin lyases (Fig. **2C**) [41]. In the a-subunit of *A. platensis *PC (molecular weight: 17.61 kDa, 162 amino acids) a single phycocyanobilin chromophore is attached to cysteine residue (Cys) 84, while in the β-subunit (molecular weight: 18.1 kDa, 172 amino acids) two chromophores are linked to Cys 82 and Cys 153 [40]. A PC hexamer thus contains in total 18 phycocyanobilin molecules. The cysteine residues for bilin attachment are found at equivalent positions in PE, APC and PC amino acid sequences of diverse species, denominated Cys 88 and Cys 178 by Apt *et al.* [37]. Conserved arginine and aspartic acid residues in close vicinity to the cysteine attachment sites also interact with the chromophores [37].

**Fig. (3):**
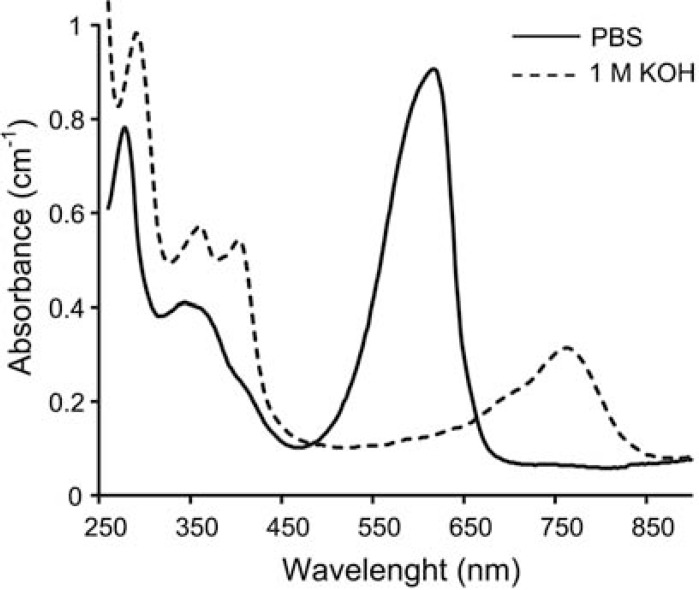
UV-Vis absorbance spectra of PC and the effect of strongly alkaline conditions. C-phycocyanin from *Arthrospira* *(Spirulina)* sp. (Sigma-Aldrich) at a concentration of 0.2 mg ml^-1^, PBS: phosphate buffered saline (pH 7.2).

In contrast to PSI and PSII proteins, phycobiliproteins are soluble and the stability of the quarternary hexameric structure is dependent on pH, ionic strength and protein concentration [42]. Phycobiliproteins of mesophilic organisms tolerate temperatures up to 47°C, but are rapidly denatured at higher temperatures [43]. Phycobiliproteins from thermophilic microorganisms exhibit higher thermostability, e.g. denaturation of *Synechococcus vulcanus* PC occurred only at temperatures above 70°C [44]. Optical spectroscopic properties of the covalently linked chromophores of biliproteins are strongly influenced by the conformation of the protein [42, 45]. When the secondary, tertiary, and quaternary structures of the protein are denatured by temperature, urea, guanidine hydrochloride or sodium dodecyl sulfate, the visible absorption band drops in intensity, simultaneously with a decrease in fluorescence and circular dichroism intensity. In the case of PC, denaturation leads to a loss of absorbance at 620 nm (first excited state) and an increase in absorbance at 360 nm (second excited state) [42]. In the native protein, phycocyanobilin is forced to adopt an extended (open) conformation due to specific interactions with amino acid side chains of the protein. In contrast, these interactions are missing when the apoprotein is denatured, allowing the chromophore to adopt a thermodynamically favored closed conformation, which in turn leads to the observed spectral changes. Strongly alkaline electrolytes which are used for enhancing the efficiency of PEC cells also lead to denaturation of PC. This is obvious from the spectral changes after exposure of hexameric PC from* Arthrospira (Spirulina)* sp. to 1 M KOH (Fig. **3**). Nevertheless, it was possible to increase the photocurrent density of hematite PEC cells that were operated with a strongly alkaline electrolyte by sensitizing the photoanode surface with *Arthrospira* PC (discussed in the section below).

PC is a promising candidate for the sensitization of PEC cells due to its spectral properties and the ease of handling (high solubility, high storage stability). However, practicability of PC as photosensitizer also depends on the price and availability of this biomaterial. In cyanobacteria, red algae, cryptomonads and glaucophytes, phycobiliproteins comprise up to 60% of soluble proteins [37]. The PC content of the major producer organism, *Arthrospira (Spirulina) *sp., can reach up to 20% of the cell dry weight [46]. *Arthrospira *is an ubiquitous microorganism and has probably been used by mankind for food purposes since ancient times [47]. The total amount of *A. platensis* biomass produced currently in a year is around 3000 t; however, the major part is not used for PC production but as whole cell product in the health food and animal feed sectors [48, 49]. PC is produced commercially with photoautotrophic cultures in outdoor open-pond or raceway systems [50]. It is mainly used as colorant for beverages, sweets and cosmetics. The annual market volume is US$10-50 million with a price of $500 per kg for food-grade PC [49, 51]. PC also has several potential therapeutic applications due to antioxidant, anti-inflammatory and anti-cancer activities [52]. PC productivities in open pond systems reach 0.024 g L^1^ d^-1^ and are limited by the low penetration depth of sunlight already at relatively low biomass concentrations in the g L^-1^ range [50]. Much higher biomass concentrations of up to 109 g cell dry weight L^-1^ are feasible in heterotrophic fed-batch cultures of the eukaryotic Rhodophyte *Galdieria sulphuraria* in bioreactors, enabling PC productivities of up to 0.86 g L^-1^ d^-1^ [50].

Production of PC as recombinant protein is also possible, but it is challenging to obtain multichain holo-phycobiliproteins, which requires synthesis of the α- and β-chains in parallel with the correct phycobilin chromophores and enzymes catalyzing chromophore attachment. Engineered, recombinant *Anabaena* sp. PC can be overexpressed from plasmids in a homologous, autotrophic host strain [53]. In the latter study the *cpcA* and *cpcB* genes encoding the PC subunits were successfully fused with DNA sequences encoding a hexahistidine tag, a streptavidin-binding tag or an oligomerisation domain for the attachment to surfaces or other proteins. Complete recombinant PC has not yet been expressed in a heterotrophic, heterologous host. However, it is possible to produce the holo-PC α-subunit of *Synechocystis* sp. PCC6803 in the standard industrial expression system *Escherichia coli *with a yield of 0.4 mg g^-1^ fresh weight [54]. In the latter study a two plasmid system was used for expression of the PC α-subunit in combination with all necessary enzymes for the conversion of heme to 3Z-phycocyanobilin (heme oxygenase 1, 3Z-phycocyanobilin:ferredoxin oxidoreductase) and the attachment to the apoprotein (heterodimeric lyase CpcE/CpcF). The yield of the holo-PC α-subunit in *E. coli* can be increased to 8 mg g^-1^ fresh weight when it is expressed as maltose binding protein fusion for solubility enhancement [55].

## BIOENGINEERED HEMATITE PEC CELLS

Hematite thin films in PEC cells do not absorb light efficiently above a wavelength of 600 nm. One approach to increase their efficiency is the DSSC concept. In the following we describe how the photocurrent density of PEC cells can be increased by sensitizing nanostructured hematite photoanodes with the light harvesting protein c-phycocyanin.

A prerequisite for the proper functioning of bio-hybrid PEC and PV devices is the tight association of proteins with the electrode surface. Proteins can be immobilized on surfaces either by adsorption, driven by hydrophobic (van der Waals) and electrostatic interactions, by entrapment in matrixes such as *in situ* polymerized hydrogels, or by the formation of covalent bonds between the polypeptide chain and chemical groups of the surface, referred to as crosslinking [e.g. 20,^ 27^,^ 28^,^ 56^-^58^]. Depending on the physico-chemical conditions prevailing in the aqueous phase, adsorbed proteins can desorb again, while covalent linkage ensures a stable immobilization [57]. Immobilization of proteins on solid supports often leads to a significantly increased stability [57, 59-61]. Adsorption of proteins to hematite has been studied by several authors and was found to strongly depend on pH, the ionic strength and the type of the protein. Human serum albumin with an isoelectric point (pI) of 5.2 adsorbs best at pH 5.0, while the maximum amount of bovine pancreas ribonuclease (pI = 9.6) per surface area adsorbs at pH 10 [62]. The observed adsorption maxima at pH values close to the pI of both proteins, i.e. when net charges are minimal, indicate that hydrophobic interactions dominate the attachment to the surface in these cases. Adsorption of cytochrome *c* from horse heart (pI = 10-10.5) to hematite (pI = 8.4) was limited to a narrow pH range between pH 8.5 and 10 that corresponds to positive charges on the protein and negative charges on hematite [63, 64]. In this case, the driving force for surface attachment seemed to be electrostatic interaction. Covalent attachment of proteins to hematite (α-Fe_2_O_3_) has not been described by others, however, various approaches for immobilization of trypsin on a related material, maghemite (γ-Fe_2_O_3_) nanoparticles, were studied in detail [65]. The authors first introduced reactive chemical groups to the surface either by grafting functional silanes (e.g. 3-aminopropyltriethoxysilane, APTES), or by reacting maghemite OH groups with N-[*p*-c>-maleimidophenyl]iso-cyanate. Trypsin was then linked covalently to the activated surface by several different reaction mechanisms in one to three additional steps. It was found that the immobilization *via* amide bonds with the side chains of lysine and arginine residues on the trypsin surface yielded the highest specific activity of immobilized biocatalyst after washing.

**Fig. (4):**
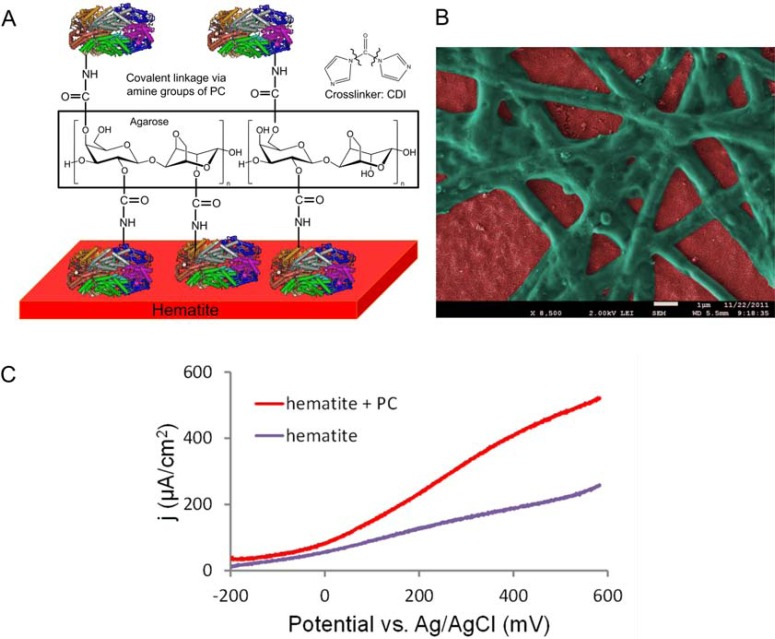
Bioengineered hematite PEC cell generated by chemical crosslinking [14]. (**A**) Scheme of hematite thin film functionalization with PC by agarose-CDI-crosslinking. (**B**) Colored SEM micrograph of a PC-coated hematite film; green: PC, red: hematite surface. (**C**) Photocurrent density enhancement achieved by PC functionalization in a hematite PEC cell; illumination: simulated solar light (AM 1.5, 100 mW cm^-2^), scan rate: 50 mV sec^-1^, electrolyte: 1 M KOH.

In our first approach for sensitizing hematite thin films with PC, a three step procedure that involved protein crosslinking via amide bonds proved to give the best results (Fig. **4A**) [14]. “Plain” coating of the PC solution on hematite yielded no reproducible photoenhancing effect, whereas the employment of a linker molecule, carbonyl di-imidazole (CDI) caused a significant, reproducible increase of photocurrent density [14]. The first step of the bio-functionalization procedure consisted of the adsorption of PC (from *Arthrobacter *sp., Sigma-Aldrich) to the hematite surface at neutral pH in phosphate buffered saline. At this pH the hematite surface is positively charged, while PC (pI = 4.64) has a net negative charge, thereby facilitating adsorption to the surface by electrostatic interactions. Next, the surface was coated with agarose and dried in order to provide a protective environment for the proteins [66]. In a third step the OH groups of the agarose were crosslinked to surface-exposed amine groups of the adsorbed PC and of additional PC supplied with the crosslinking solution. The crosslinker CDI is an active carbonylating agent that contains two imidazole leaving groups (Fig. **4A**). Upon PC-agarose-CDI treatment, a structured surface with web-like appearance was obtained, presumably composed of chains of immobilized proteins and regions of bare hematite in between (Fig. **4B**). In a hematite PEC cell with 1 M KOH as electrolyte, this bio-functionalization increased the photocurrent density from 281 µA cm^-2^ (bare hematite) to 491 µA cm^-2^ under simulated solar illumination (AM 1.5) (Fig. **4C**) [14]. Interestingly, the strongly alkaline electrolyte (1 M KOH, pH 13.6) did not disturb the photoenhancing effect of immobilized PC, although PC in solution denatures under similar conditions (Fig. **3**). This can be explained either by a stabilizing effect of the agarose-embedding and CDI crosslinking or by a photosensitizing effect of the phycobilin chromophore alone, which still absorbs light even when PC is denatured (Fig. **3**). The immobilized PC apparently also did not suffer from rapid degradation (photobleaching) because the photocurrent remained stable for at least one hour under continuous illumination with simulated solar light [14]. The photoenhancing effect was also evident without hematite. FTO electrodes with adsorbed PC yielded higher photocurrents than pristine FTO, although photocurrent densities were generally 10 times lower compared to hematite electrodes [14]. The mechanism of the photoenhancing effect is not yet fully understood. Incident photo-to-charge carrier efficiency (IPCE) measurements of pristine and PC-coated hematite photoanodes indicate that the enhanced photocurrent is in fact due to extra light harvesting of PC, in particular at wavelengths above 600 nm. Possibly, excited state phycocyanin directly injects electrons into the conduction band of hematite, as has been suggested for PC adsorbed to colloidal TiO_2_ nanoparticles [20]. Alternatively, an indirect mechanism could involve the photogeneration of hydroxyl radicals by PC (extra water oxidation), which in turn act as electron scavengers at the hematite surface.

**Fig. (5):**
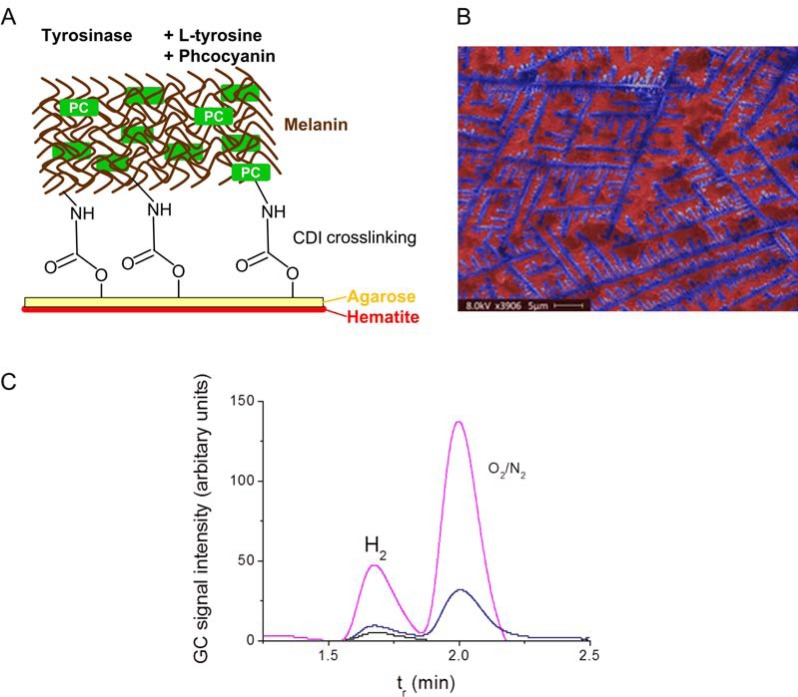
Bioengineered hematite PEC cell with PC-melanin coating. (A) Scheme of tyrosinase-catalyzed melanin formation and crosslinking with PC and agarose. (B) Colored SEM micrograph of PC-melanin coated hematite showing a comb-like, self-similar pattern; blue: PCmelanin structures, red: hematite surface. (C) Increased hydrogen and oxygen formation achieved by PC-melanin coating of hematite; GC chromatograms of headspace samples of PEC cells after 20 min operation, electrolyte: PBS pH 7.2, external overpotential: 1.0 V vs. Ag/AgCl reference electrode, blue line: pristine hematite photoanode, pink line: PC-melanin functionalized hematite photoanode, black line: reference chromatogram of H2 standard.

**Fig. (6):**
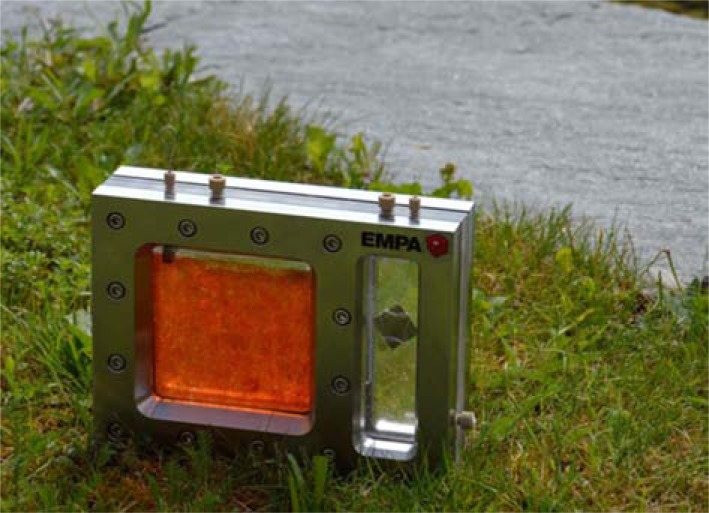
BioPEC solar hydrogen generator with a hematitephycocyanin hybrid photoanode (red pane, size: 10 x 10 cm). The hematite layer was deposited on standard fluorine doped tin oxide coated glass. Electrolyte: PBS solution in contact with a platinum counter electrode.

Crosslinking of proteins to surfaces with highly reactive chemical compounds may lead to inactivation due to undesired modifications, e.g. of active site residues [65]. Enzyme-based crosslinking strategies represent a milder method with the potential to obtain higher amounts of active protein per surface area [58]. A generic enzymatic crosslinking method that is not dependent on specific recognition sequences (peptide tags) utilizes tyrosinase. Tyrosinases perform a two-step oxidation of L-tyrosine and other mono-phenols to reactive quinone species at the expense of molecular oxygen as co-substrate [67]. The final product of enzymatic L-tyrosine oxidation, L-DOPA quinone, spontaneously polymerizes and forms the brown-black pigment melanin, which is an important UV-protecting biopolymer found in all kingdoms of life [68, 69]. L-tyrosine residues exposed at the surface of proteins can also be activated by tyrosinase and in turn react with amine and thiol groups of other proteins or polymers in a Michael-type addition [58, 70, 71]. Quinone-groups of polyphenols generated by tyrosinase catalysis from added monomers can also react with these functional groups, thereby incorporating proteins in a polymeric network. In our laboratory we successfully used recombinant bacterial tyrosinase from *Verrucomicrobium spinosum* for the synthesis of crosslinked enzyme aggregates (CLEAs) of the industrially relevant biocatalyst *Candida antarctica* lipase B [67, 72]. Efficient CLEA formation was only observed when a monomeric tyrosinase substrate (phenol) was added to the reaction mixture which polymerized and acted as a crosslinking stimulus. We applied this procedure for the immobilization of PC on hematite, but with L-tyrosine instead of phenol as monomer. The rational was to combine a gentle, covalent immobilization of PC with enhanced electronic coupling to the electrolyte and hematite due to the formation of a melanin matrix. Melanin can be considered as a disordered organic semiconductor, and natural melanins also contain embedded proteins [73, 74].The chemo-enzymatic PC-melanin functionalization procedure consisted of three steps: (i) coating of the pristine hematite surface with agarose, (ii) reaction with CDI to generate reactive sites for enzymatic crosslinking, (iii) enzymatic melanin formation and crosslinking of PC by adding a mixture of L-tyrosine, PC and tyrosinase to the surface (Fig. **5A**) [75]. Interestingly, after the enzymatic reaction the organic layer was arranged in a regular, self-similar pattern with a comb-like architecture on the hematite surface (Fig. **5B**). High resolution SEM images indicated that comb branches were aggregates of globular primary particles of around 200 nm diameter. The photocurrent density of PC-melanin-functionalized hematite PEC cells with PBS as electrolyte was three fold higher than that of pristine PEC cells. The maximal photocurrent density reached with PC-melanin-hematite photoanodes under simulated solar illumination (AM 1.5) was 450 µA cm^-2^ (with an overpotential of 1 V versus an Ag/AgCl reference electrode). Melanin coating alone lead to a slight decrease instead of an increase 

in photocurrent density, indicating that PC is a crucial component for photocurrent enhancement. No photoenhancing effect of PC-melanin functionalization was observed when PEC cells were operated with 1M KOH electrolyte. This is probably due to dissolution of the coating, as melanin is known to be unstable under alkaline conditions. The increased photocurrent density of PC-melanin-functionalized hematite films corresponded to an increased rate of H_2_ and O_2_ evolution of PEC cells, as evident from analysis of the headspace by gas chromatography (GC, Fig. **5C**). PC-melanin-hematite PEC cells can be operated with phosphate buffered saline. The use of such an environmentally benign, non-toxic, non-corrosive and pH-neutral electrolyte instead of 1M KOH could be important for the integration of solar hydrogen production utilities or appliances in residential homes. We already scaled up the fabrication of PC-melanin-coated, nanostructured hematite photoanodes to 100 cm^2^ units and constructed a demonstrator for longer-term use tests (Fig. **6**). The longer-term stability of PC-functionalized hematite PECs over several days, months or even years has not yet been studied. It is possible that reactive oxygen species generated during the photocatalytical water splitting reaction destroy the immobilized PC over time. Further optimized immobilization methods which stabilize the PC structure might overcome this problem. 

## CONCLUSIONS AND OUTLOOK

The new possibility of fabricating bio-hybrid hematite photoanodes for PEC cells raises many further questions and leaves room for further improvements. The relevance of the observed nano-structured patterns of the organic coatings for photocurrent increase is not yet understood. Also the opto-electronic interaction of melanin, PC and hematite awaits further elucidation. In order to reduce the costs for the bio-photosensitizer one could evaluate the use of crude *A. platensis *cell extracts instead of purified PC for the coatings. A simpler bio-functionalization procedure is also desirable, for example by engineering PC for strong, oriented binding to hematite which possibly renders agarose coating superfluous. A possibility for a further increase in the efficiency of bio-engineered PEC cells could be the immobilization of hydrogen-forming biocatalysts on the cathode (e.g. oxygen-tolerant hydrogenases). Finally, a key topic which needs to be addressed before such systems can get closer to real application is the stability of immobilized light-harvesting proteins on PEC electrodes under long-term, periodic illumination.

## Figures and Tables

**Table 1.** Best-performing protein-functionalized PEC and PV cells for five different photoanode materials, n.a.: not analyzed. The intensity of solar radiation reaching the earth’s surface is 89 mA cm-2 (sea level, sun in zenith, clear sky, moderate humidi-ty and aerosol concentration) [22]. Fold increase: Relative increase in photocurrent density by immobilized photosynthetic proteins. Efficiency: conversion of input radiation energy to electrical energy.

Electrode material	Photosynthetic protein(s)	Immobilization (crosslinker)	Electrolyte	External potential a	Light intensity		Photocurrent density (max.)		Efficiency (max.)	Ref.
				[V]		[mW cm-2]	[µA cm-2]	Fold increase	[%]	
TiO2	Bacteriorhodopsin *H. salinarum*	Covalent (3-mercapto-propionic acid)	1 M citrate buffer pH 7	0.64		100 (AM 1.5) b	650	1.5 x	0.42	[21]
TiO2/WO3	Photosynthetic reaction center, *Rb. sphaeroides*	Adsorption	Tris-HCl pH 8.0 + 8 mM NaS2O4	0.1		5	30	5 x	0.06	[23]
TiO2/ZnO	PSI, *T. elongatus*	PsaE PSI subunit engineered with ZnO binding tag	0.5 M Co(II) complex, 0.05M Co(III) complex, 0.2 M LiClO4 , org. solv. c	0		100 (AM 1.5) b	362	4 x	0.08	[24]
p-doped silicon	PSI, spinach	Adsorption	100 mM KCl + 0.2 M methyl viologen	0.28		190 (≥633 nm)	875	4 x	0.13	[25]
a-Fe2O3	C-phycocyanin *Arthrobacter*	CDI-agarose crosslinking	1 M KOH pH 13.6	0.5		100 (AM 1.5) b	491	2 x	0.25	[14]
ITO	PSII, *T. elongatus*	Adsorption	80 mM K, Ca, Mg chloride, 1 mM DCBQ d, MES e pH 6.5	0.5		8	22	n.a.	0.14	[26]
Gold	PSI, *T. elongatus*	Incorporation in Osmium-redox hydrogel	Na citrate pH 5.5, 10 mM MgCl2, 10 mM CaCl2, 2 mM methyl viologen	0		1.8 (680 nm)	33	n.a.	0.31	[27]

a applied external overpotential (bias) against Ag/AgCl reference electrode (standard hydrogen electrode)

b simulated solar light corresponding to the average air mass coefficient (AM) of the Earth's atmosphere

c organic solvent, 60‰ ethylene carbonate and 40% acetonitrile (v/v)

d 2,6-dichloro-1,4-benzoquinone

e 2-(N-morpholino)ethane sulfonic acid buffer system
